# Insights into the assembly mechanisms, co-occurrence patterns, and functional roles of bacterial community in an estuary system

**DOI:** 10.1128/spectrum.02249-25

**Published:** 2026-08-17

**Authors:** Yongjie Wu, Yang Zhang, Zhiwen Luo, Cheng Wang, Changdong Ke, Kaiming Li, Bixian Mai, Renren Wu

**Affiliations:** 1 State Environmental Protection Key Laboratory of Water Environmental Simulation and Pollution Control, South China Institute of Environmental Sciences, Ministry of Ecology and Environment of the People’s Republic of China, Guangzhou, People’s Republic of China; 2 School of Environmental Science and Engineering, Sun Yat-sen University 26469 , Guangzhou, China; 3 State Key Laboratory of Organic Geochemistry and Guangdong Key Laboratory of Environmental Resources Utilization and Protection, Guangzhou Institute of Geochemistry, Chinese Academy of Sciences 74556 , Guangzhou, China; University of Illinois Urbana-Champaign, Urbana, Illinois, USA

**Keywords:** estuary, biogeography, functional potential, community assembly, water, sediment

## Abstract

Microbial drivers of biogeochemical cycles in estuaries remain understudied, particularly regarding biodiversity-function relationships. Using 16S rDNA sequencing, we analyzed taxonomic and functional profiles of bacterial communities in the water and sediment along the salinity gradient in Zhenhai Bay (South China). Significant differences in diversity, structure, composition, and function were observed between water and sediment habitats, indicating habitat influence. The terrestrial pollution sources detected in this study have a limited contribution to Zhenhai Bay, with unknown sources accounting for the majority of the total pollution, indicating the complexity of the estuarine environment in Zhenhai Bay. Additionally, most of the functional indicators of C, N, and S cycling were more enriched in the sediment, suggesting a more active biochemical cycle in the sediment than in the water. The spatial turnover of bacterial community composition in the water was much faster than the functional potential, and it showed higher functional redundancy in the water than in the sediment. Furthermore, except for the taxonomic community composition in the water, the functional community in the water and the taxonomic and functional community composition in the sediment were all dominated by stochastic processes. This indicated that strong environmental filtering of the taxonomic community in the water was due to hydrographic conditions or undetected environmental stressors. Overall, the results of this research provide a reasonable characterization of the taxonomy and function of microbial communities in an estuary and provide insights into how spatial changes in the taxonomic and functional community alter the nature and stability of microbial networks.

**IMPORTANCE:**

Understanding how bacteria drive essential processes like carbon and nutrient cycling is crucial for managing sensitive estuarine ecosystems. However, we lack clear knowledge of how the diversity of these microbes diversities relate to their actual functions in complex, real-world settings like Zhenhai Bay. This study provides a comprehensive picture of the identity and functional roles of bacterial communities across different habitats (water vs sediment) within a dynamic estuary. We found distinct patterns: sediment hosts more active nutrient cycling, water communities show greater functional flexibility, and pollution sources are surprisingly complex and largely unidentified. Critically, we show that the assembly of these communities is often governed by randomness, except in water, where environmental forces strongly filter which bacteria survive. These insights help predict how estuarine ecosystems might respond to pollution or change, improving management strategies to protect their health and the vital services they provide.

## INTRODUCTION

Estuaries are dynamic environments where nutrient-rich river waters mix with shallow coastal waters and are particularly sensitive to terrestrial pollution inputs, resulting in significant changes in ecosystem structure and function (1). The unique environmental characteristics of the estuary make it one of the most vulnerable aquatic ecosystems, and the sustainable development of estuarine ecosystems depends on the maintenance of favorable environmental conditions and the continued functioning of associated ecological processes, particularly those involving microbial communities (2–5). Estuaries surrounding densely populated cities are particularly vulnerable to anthropogenic disturbance events that can severely impact estuarine processes and disrupt the vital ecosystem services that estuaries provide, such as carbon storage, habitat for unique communities, nutrient cycling, aquaculture, and wild fisheries (2, 6, 7).

Microbiomes play an important role in estuarine ecosystem functions, such as regulating nutrient cycling, and may provide an important indication of estuarine ecological health (2, 3, 8–10). However, linking the dynamics of complex estuarine microbial communities to ecosystem-scale biogeochemical processes remains a challenge in the field of microbial ecology. Despite a large number of studies on microbial communities in estuaries, no consistent conclusions have yet been reached (2, 3, 11, 12). Previous studies on estuarine microbiomes have investigated microbial diversity, structure, and composition; microbial biogeography (5, 13, 14) and seasonal variability (15); metabolic capabilities and functional genes (2, 16–18); and the connectivity and stability of microbial communities in different environments (19–23). However, the associated mechanisms of microbial community assembly under complex natural and anthropogenic influences remain unclear.

Deterministic (e.g., species traits, abiotic environmental filtering, and biotic interactions) and stochastic processes (e.g., birth, death, dispersal, and speciation rates) can explain the variation in microbial community assembly (24, 25). The assembly and biogeography of bacterial communities in estuarine ecosystems are controlled by environmental factors (26), with water mass, temperature, pH, nutrient concentration, and salinity all identified as important drivers of community assembly processes (26–30). On the contrary, researchers also found that stochastic processes were more important than deterministic processes in structuring microbial communities in estuarine sediments (7, 21). Such discrepancies could be explained by differences in spatial scale and environmental gradients (31, 32). Riverine inputs will create salinity, nutrient, and hydrodynamic gradients in estuaries, which influence the deterministic and stochastic processes of community assembly in estuaries (33–35). However, it is unclear how microbial community assembly in estuarine water and sediments occurs.

A previous study showed that the key level at which to address the assembly and structure of microbial communities may not be taxonomy, but rather the more functions (36). This can mean that the microbial functions were widely distributed across a variety of taxa or phylogenetic groups; this is called functional redundancy. Functional redundancy is used to maintain ecosystem functions in the face of disturbances that could lead to the disappearance of local species, a gap that can be filled by species with comparable functions but different taxonomic classifications and habitat preferences (36, 37). Therefore, it is essential to elucidate the microbial dynamics mechanism of how contrasting water and sediment shape the microbial composition and function in estuarine ecosystems under complex environmental constraints.

Understanding estuarine microbial composition and function is critical for effective management of estuarine and marine ecosystems. While many studies have addressed this topic, comparative studies of water and sediment habitats are lacking, resulting in a lack of consistent and reliable patterns of estuarine microbial communities. To address this gap, we conducted an integrated analysis of 16S rDNA sequencing data from water and sediment in Zhenhai Bay. Zhenhai Bay is located in the marine area of Jiangmen City, Guangdong Province (21°41′–21°56′30″N, 112°21′–112°32′E). The area covers about 100 km^2^ and is a typical trumpet-shaped estuary. Upstream of the area is the largest mangrove wetland park in the Pearl River Delta region, Guangdong Zhenhai Bay Mangrove Wetland Park. The central and southeastern parts are important oyster farming areas in Guangdong Province, while the southwest side of the estuary is a coastal tourist resort. Zhenhai Bay predominantly drains mainly rural pastoral catchments and is therefore affected by anthropogenic activities. The most polluted area along the Zhenhai Bay estuary is nitrogen and carbon pollution. Therefore, this area provides an ideal natural laboratory for understanding the composition, function, and assembly of the estuarine bacterial community and its variation along environmental gradients. In this study, the bacterial community in water and sediments was investigated at 10 sites along the Zhenhai Bay estuary. In detail, three specific objectives were pursued: (i) to characterize the ecological traits of bacterial taxonomic communities and their potential functions in the context of the estuary; (ii) to investigate the influences of terrestrial pollution on the Zhenhai Bay estuary; and (iii) to evaluate the forces that determined the assembly process and functional redundancy of these microbiomes. The results of this research could lead to a better understanding of how estuaries respond to increasing terrestrial pollution loads via microbial processes and motivate the restoration of the estuary.

## MATERIALS AND METHODS

### Sample collection and geochemical analysis

**Fig 1:**
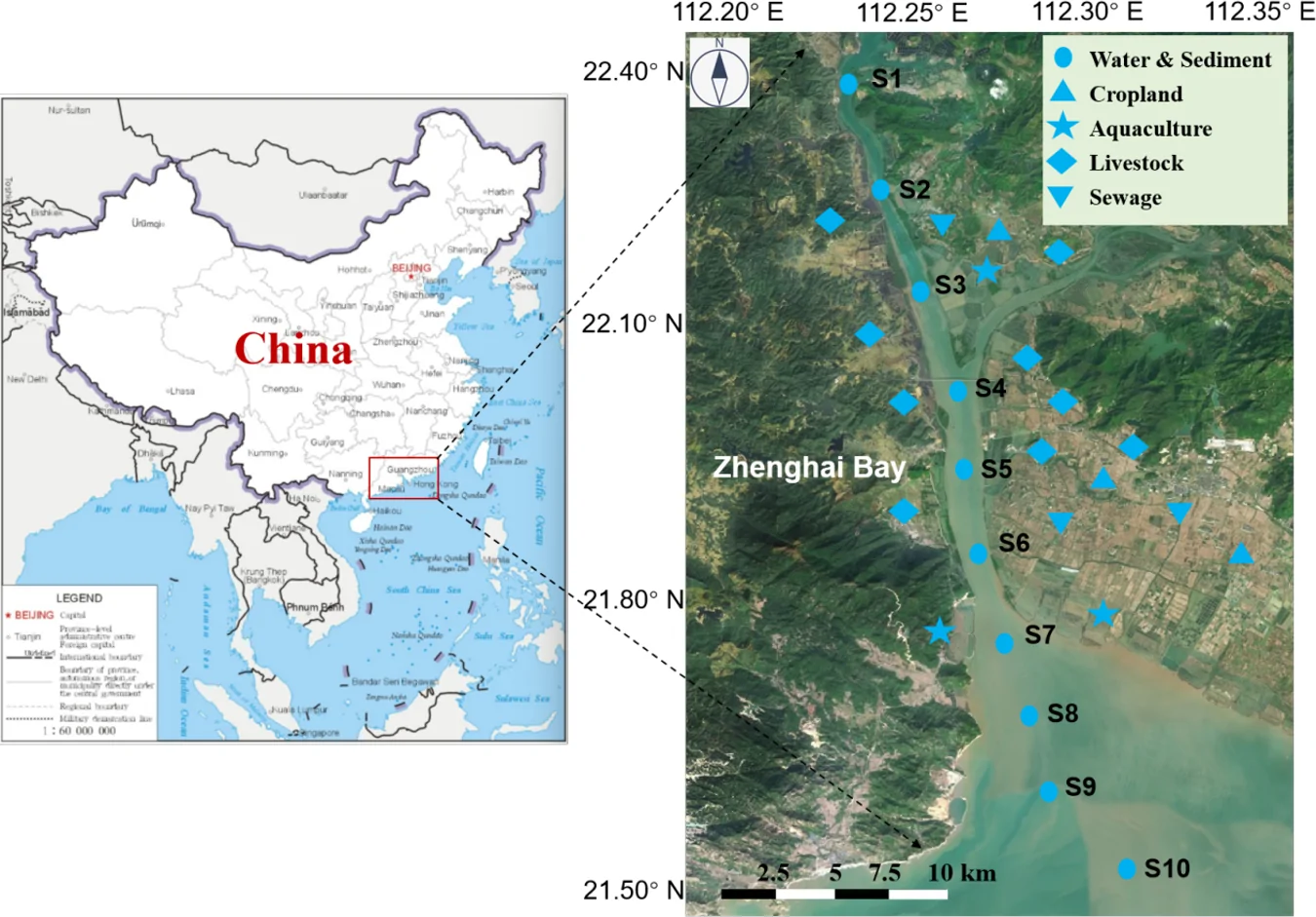
Maps showing the sampling stations along the Zhenhai Bay estuary.

Water and sediment (0–5 cm) samples were collected in triplicate at 10 sampling sites (S1–S10) along the Zhenhai Bay estuary (Fig. 1) in August 2021. *In situ* measurements of dissolved oxygen (DO), salinity, pH, and conductivity were performed using a portable instrument (EXO2; YSI, USA). Ammonium and nitrate were extracted from the sediments with 2 M KCl and measured using a continuous flow analyzer. Total nitrogen and total phosphorus (TP) in water and sediment samples were also measured using standard methods. All analyses were performed on water or sediment samples in triplicate. In addition, to investigate the influence of terrestrial pollution on the Zhenghai Bay estuary, 18 livestock, 9 soil of the cropland, 18 aquaculture water, and 9 sewages (wastewater treatment plants) were sampled throughout the watershed and stored with the filters under the same conditions (Fig. 1).

### DNA extraction and sequencing

Extraction and purification of microbial community DNA from filtered water, sediment/soil, and feces were performed using the Power Water DNA Isolation Kit, Power Soil DNA Isolation Kit, and Power Fecal DNA Isolation Kit, respectively (MOBIO Laboratories, Carlsbad, CA, USA). DNA quantity and quality were determined using a NanoDrop spectrophotometer (NanoDrop Technologies Inc., Wilmington, DE, USA), and the highest quality DNA was used for library construction. A dual-index sequencing strategy was used to amplify the V3 and V4 regions of the bacterial 16S rDNA gene using universal primers 338F (5′-ACTCCTACGGGAGGCAGCA-3′) and 806R (5′-GGACTACHVGGGTWTCTAAT-3′) (38). Amplification conditions were as follows: initial denaturation at 95°C for 5 min, followed by 30 cycles of 95°C for 30 s, 52°C for 30 s, and 72°C for 30 s, and ending with a final extension at 72°C for 10 min. Library quality was assessed using a fragment analyzer, and libraries were subjected to 250 bp paired-end sequencing on a HiSeq platform from Novogene Technologies Corporation (Beijing, China). The microbial data were deposited in the NCBI SRA under accession numbers PRJNA1188316 and PRJNA1342806, making the data publicly available for future research and analysis.

### Statistical analysis

All analyses were performed on thorium-normalized data and mean triplicate for chemical data. For sequencing data, the mean of triplicates for each sample was used to generate the amplicon sequence variant (ASV) table. Demultiplexed reads were trimmed of primer binding sequences using Cutadapt, and reads with no primer match were discarded (39). Trimmed reads were then processed in USEARCH to generate ASV count tables (40). An ASV table including all samples was normalized with 37,996 reads per sample for downstream analyses. Singletons were removed, and ASV tables were constructed using the usearch_global command with a similarity threshold of 99%, and taxonomies were inferred from the Silva (v138.1) database for 16S rDNA gene sequences (41). Ecological functions were performed in Galaxy/DengLab using the FAPROTAX with default parameters (42).

Bacterial alpha diversity (including Shannon diversity, richness, Simpson diversity, and Chao1 diversity) was measured using USEARCH (43). We used Spearman correlation analysis to assess the relationship between taxonomic communities or potential functions and environmental factors. Analysis of variance and *t*-tests were performed using GraphPad Prism 8.0 to assess differences and check significance levels at *P* < 0.05. Principal component analysis (PCA) was performed using the vegan package based on Bray-Curtis dissimilarity. Visualization of the distance decay relationship was based on community similarity calculated using a Bray-Curtis index after normalization of the data set by cumulative sum scaling using the metagenomeSeq package (44). Geographical distance was measured using a “Vincenty” (ellipsoid) great circle distance to account for the curvature of Earth, relying on the enmSdm and geosphere packages (45).

Source-tracking analysis was used to estimate the influences of terrestrial pollution inputs to Zhenhai Bay using a Bayesian approach, which may imply the contribution of dispersal sources to the Zhenhai Bay microbiome (46, 47). Samples from the water and sediment in Zhenhai Bay were separately designated as sinks, and the terrestrial pollution samples were designated as sources. Taxonomic communities and functional potentials were visualized using the R package “pheatmap” with the Kruskal-Wallis test to compare the differences between water and sediment. Redundancy analysis (RDA) was employed to discuss the relationship between environmental factors and microbial compositions. We constructed a random forest model to correlate the habitat types (water and sediment) using taxonomic data at the genus level and functional profile data. Random forest models were constructed using the R package “randomForest.” Model significance and cross-validated *R*^2^ values were assessed with 1,000 permutations of the response variable using the “A3” package. Tenfold cross-validation was performed using the “rfcv()” function to select appropriate features (48). The “varImpPlot” function was used to show the importance of the features used in the classification. A pairwise indicator species analysis was used to identify the indicators of bacterial genera and functions (from functional predictions) that were significantly associated with the habitat type for each contrast. This was implemented in the R package “labdsv.” The IndVal score for each bacterial genus and predicted function was the product of the relative abundance and mean abundance in water and sediment, and significance was calculated by random reassignment of groups (1,000 permutations). Biomarker taxa or predicted functions were defined as the intersection of important taxa or predicted functions screened by random forest, and indicator taxa or predicted functions screened by indicator species analysis (48).

Only ASVs with an average relative abundance >0.01% were kept as more precise and reliable results for subsequent network analysis (49). Taxonomic and functional potential microbial networks were constructed based on the Pearson correlation coefficient (*P* value ≤ 0.05) of log-transformed ASV abundances on the Molecular Ecological Network Analyses Pipeline (http://ieg2.ou.edu/MENA/) (50, 51). The network was visualized using Gephi software. Additionally, we identified the keystone nodes for each community by assessing their within-module connectivity (Zi) and among-module connectivity (Pi) (52). Four types of nodes were identified as potential keystones: (i) module hubs (highly connected nodes within modules with Zi > 2.5 and Pi ≤ 0.62), (ii) network hubs (highly connected nodes within the entire network with Zi > 2.5 and Pi > 0.62), (iii) connectors (nodes that connect modules with Zi ≤ 2.5 and Pi > 0.62), and (iv) peripherals (nodes with Zi ≤ 2.5 and Pi ≤ 0.62) (50). To determine the potential contribution of stochastic processes to microbial community assembly, the neutral community model (53) was performed to understand the stochastic and deterministic processes on the assembly of microbial communities. The neutral community model was conducted on Tutools platform (https://www.cloudtutu.com/chartOverview).

## RESULTS

### Geochemical characteristics of the water and sediment samples

The physicochemical properties of the water and sediment samples used in this study are summarized in Table S1. Environmental parameters varied widely between the water and sediment environments. The most significant gradient between these two sampling sites was salinity in both water and sediment, and water geochemical parameters varied more greatly between different samples than in the sediment. The sediment geochemical parameters were varied more stably along the salinity gradient, and the concentrations of T, TP, NH_4_^+^, NO_3_^−^, and TN were all decreased with increasing salinity in the water. This suggests that the water was more sensitive to the environment, such as the terrestrial pollution inputs and tides.

### Biogeography patterns of bacterial communities

In total, we generated 33,554 bacterial ASVs in the water and sediment sampled at the 10 sites. The observed alpha diversities of the bacterial community were highly similar (*P* > 0.05; Shannon, Simpson, and Chao1 diversity) between water and the sediment habitats, but the richness in the sediment was significantly (*P* < 0.05) higher than in water (Fig. S1). The six most abundant phyla accounted for >90% of the detected sequences, and their abundance varied between water and sediment (Fig. S2A). The most abundant were Firmicutes (34.46%), followed by Proteobacteria (24.32%) and Bacteroidetes (13.51%) in water, whereas Proteobacteria (33.09%), Actinobacteria (27.13%), and Cyanobacteria (11.22%) in sediment. At the genus level, the variation in microbial composition between water and sediment was much more different than at the phylum level (Fig. S2B). Furthermore, the functional distribution was similar to the taxonomic groups, showing significant differences between water and sediment (Fig. S3A and B). Functional microorganisms associated with the carbon cycle are more abundant in the water than in the sediment, and the opposite is true for the sulfur cycle, where they are more abundant in the sediment than in the water. While functional microorganisms associated with the aerobic nitrogen cycle were more abundant in the water than in the sediment, functional microorganisms associated with the anaerobic nitrogen cycle were more abundant in the sediment than in the water (Fig. S3B).

**Fig 2:**
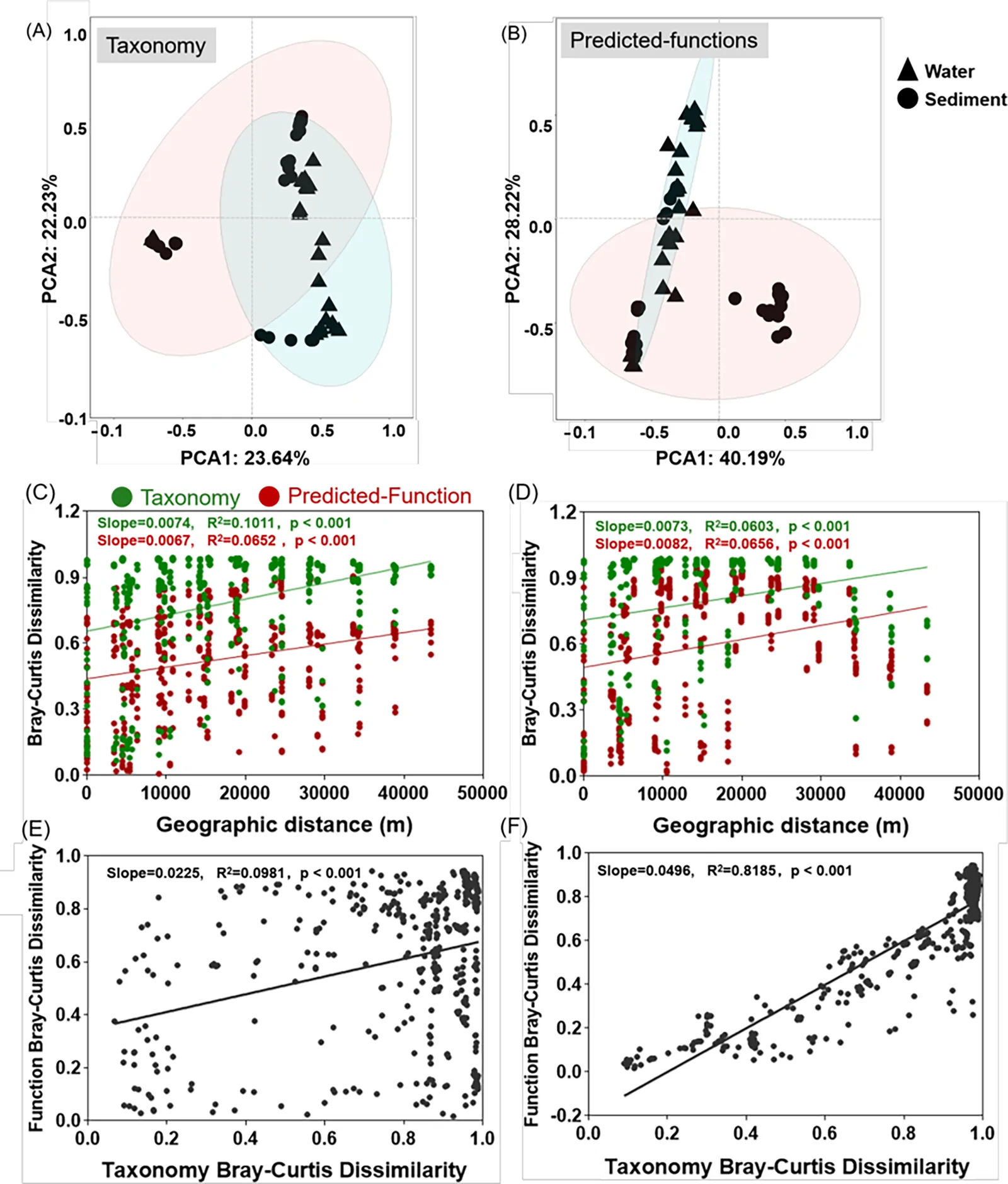
Principal component analysis for environmental heterogeneity between water (**A**) and sediment (**B**) based on the environmental factor data. Distance-decay effects in microbial compositions and predicted functions. Relationship between geographic distance (m) and bacterial community (green point) or predicted functions (red point) dissimilarity (based on Bray–Curtis distances) in water (**C**) and sediment (**D**), respectively. The relationship between bacterial community dissimilarity and predicted functions dissimilarity in water (**E**) and sediment (**F**), respectively. The solid line indicates the fitted linear regression model. Note that much steeper slopes for taxonomy than slopes for predicted functions reflect the functional redundancy of the microbial community.

PCA using Bray–Curtis dissimilarity was used to identify the bacterial compositions and potential functions of the water and sediment samples (Fig. 2A and B). Both the bacterial compositions and potential functions showed clear habitat groups. The first two principal components explained 45.87% of the variance in bacterial compositions and 68.41% of the variance in potential functions, indicating that bacterial functions were more sensitive to habitat type than compositions. To explore the distribution of microbial communities over spatial distances in water and sediment, distance-decay relationships were estimated as the slopes of ordinary least-squares regressions between geographic distances and bacterial community and predicted function dissimilarity (based on Bray-Curtis distances). Taxonomy and predicted function showed similar spatial distance decay relationships in both water and sediment, with significant (*P* < 0.001) differences in decay rates, and taxonomy in water showed the strongest distance decay than others (Fig. 2C and D). It indicates a distance decay effect on the taxonomic and functional composition of the bacterial community, and taxonomy in water was significantly impacted by distance. The slope of the relationship between taxonomic and predicted functional dissimilarities in the sediment was higher than that in the water (Fig. 2E and F), indicating that microbial communities in water may have a higher degree of functional redundancy.

### Key bacterial taxa and functions in water and sediment

**Fig 3:**
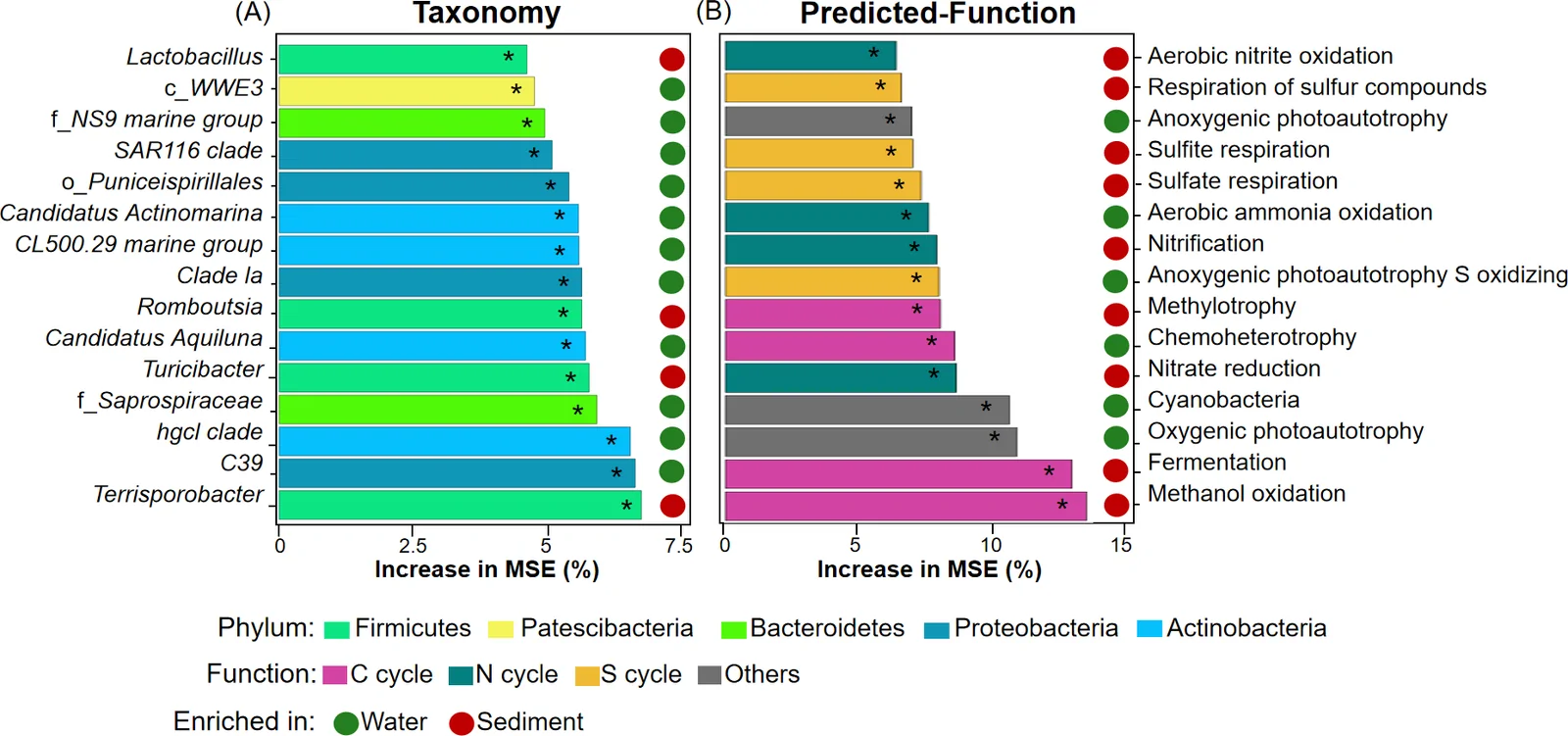
The indicator taxa and predicted functions depending on habitats. Bacterial taxonomy at the genus level and predicted functions enriched in water and sediment according to the result of IncMSE% score. The top 15 bacterial genera (**A**) and top 15 predicted functions (**B**) were identified by applying random-forest classification of the relative abundance of the microbiota and predicted functions against water and sediment.

**Fig 4:**
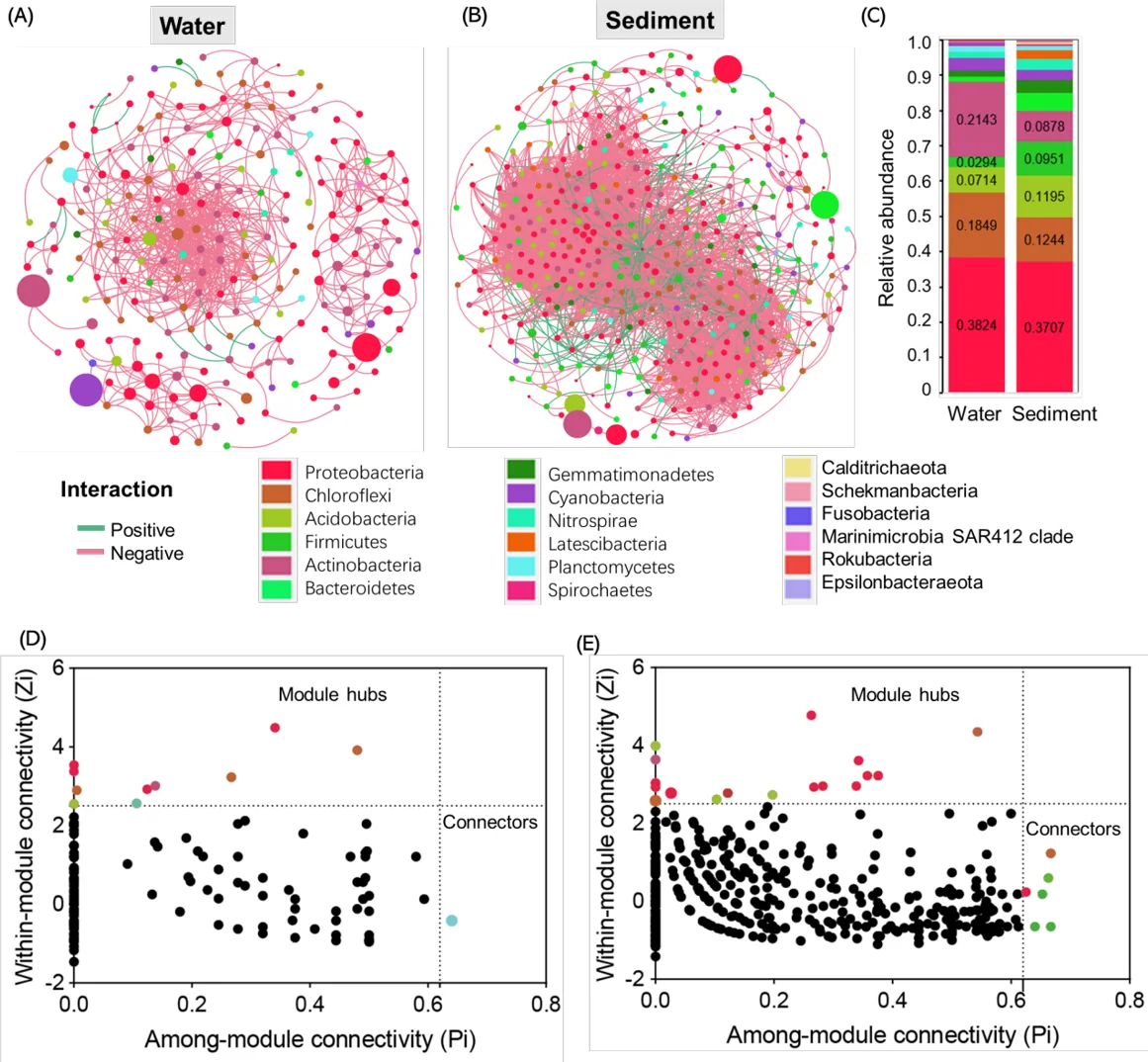
Visualized molecular ecological networks of water (**A**) and sediment (**B**) in Zhenghai estuary, and composition of bacterial communities in water and sediment (**C**). Z-P plot showing the distribution of bacterial ASVs based on their topological roles (**D and E**). The node colors represent different microbial phyla. Red lines represent negative edges (such as competition, predation, and parasitism), while green lines represent positive edges (such as commensalism and mutualism). The node size corresponds to relative abundance.

Random forest modeling was performed to assess the bacterial taxonomic and predicted functional signatures between water and sediment. Figure 3 shows the top 15 genera and functions sensitive to the habitat type. Four genera (i.e., *Terrisporobacter*, *Turicibacter*, *Romboutsia*, and *Lactobacillus* belonging to the Firmicutes) were more abundant in sediment than in water. The other 11 genera (i.e., *C39*, *hgcl clade*, f_*Saprospiraceae*, *Candidatus Aquiluna*, *Clade la*, and *CL500.29 marine group*) were more abundant in water than in sediment. There were four potential functions, each related to the carbon, nitrogen, and sulfur cycles, and three potential functions related to the other function. Nine potential functions (i.e., methanol oxidation, fermentation, nitrate reduction, methylotrophy, sulfite respiration, sulfate respiration, aerobic nitrite oxidation, and respiration of sulfur compounds) were enriched in sediment, and the other six potential functions (i.e., oxygenic photoautotrophy, cyanobacteria, chemoheterotrophy, anoxygenic photoautotrophy S oxidizing, aerobic ammonia oxidation, and anoxygenic photoautotrophy) were enriched in water. The co-occurrence networks were constructed to evaluate the co-occurrence between water and sediment microbial communities in the Zhenhai Bay estuary. The microbial community network was constructed using Spearman’s correlation coefficients (Fig. 4; Table S2). The empirical networks for microbial communities showed a higher average clustering coefficient, harmonic geodesic distance, and modularity compared with their random networks (Table S2), indicating that the cooccurrence network of microbial communities in the estuary showed a non-random modular structure. This structure was primarily influenced by the taxonomic similarity of the coexisting species. Network complexity was found to be lower for the water community compared to the sediment community, with the average degree for the water and sediment networks being 5.191 and 25.041 (Table S2), respectively. Further examination of the networks of these communities showed that the microbial community in both water and sediment was characterized by negative links (Fig. 4A and B). Most of the network nodes were mainly composed of Proteobacteria, Chloroflexi, Acidobacteria, Firmicutes, and Actinobacteria. The co-occurrence network was tighter, and more species were involved in community building in sediment samples. Additionally, potential keystones in the estuarine microbial communities were characterized using the values of within-module connectivity (Zi) and among-module connectivity (Pi). There were more potential keystone taxa in the sediment community than that in the water community (water vs sediment: 11 vs 23; Table S3). These results showed that these water and sediment microbiomes had unique network configurations and topological roles for each ASV and potential keystone taxa. A correlation analysis between the keystones and environmental factors was performed to further explore the environmental drivers in our data set.

### The balance between stochastic and deterministic processes in shaping the bacterial community

**Fig 5:**
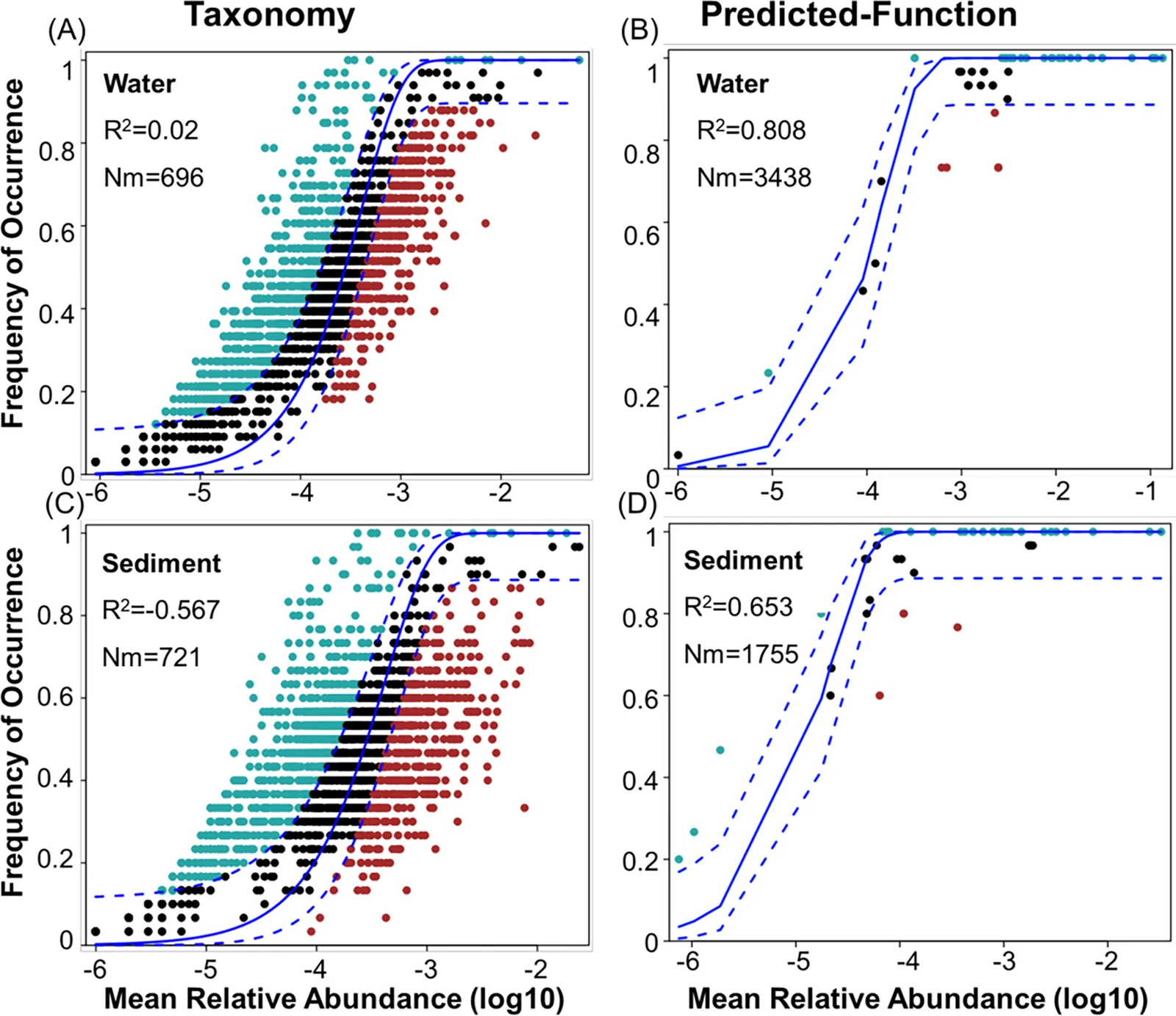
Community assembly mechanisms governing microbial taxa and functional traits. Stochastic ratio analyses using a null model showing the relative contribution of stochastic and deterministic processes in the assembly of taxonomic and functional compositions in water (**A and B**) and sediment (**C and D**), respectively. The predicted occurrence frequencies for N-cycling functional traits and taxonomic groups communities were analyzed. The model prediction has 95% confidence intervals around it, represented by the dashed blue lines. Community compositions that occurred more or less frequently than predicted by the NCM are shown in different colors.

Neutral model and niche breadth analyses were conducted to evaluate the role of stochastic-based (neutral) and deterministic-based (niche) processes in structuring the water and sediment bacterial community. The *R*^2^ values (2%, 80.8%, 56.7%, and 65.3% for the water and sediment taxonomic and functional composition, respectively) indicated that the functional community assemblages were well described by neutral-based models, and the stochastic processes made important contributions to shaping the assembly of functional communities in both water and sediment (Fig. 5A and B). Neutral community model analysis showed that the assembly of functional communities in the Zhenhai Bay estuary was influenced by stochastic processes (*R*^2^ = 0.808 for microbiomes in water samples, respectively, and *R*^2^ = 0.653 for the corresponding sediment microbiomes; Fig. 5). The taxonomic community in the sediment was shaped by both deterministic and stochastic processes (*R*^2^ = 0.567), while the taxonomic community in the water was shaped by deterministic processes (*R*^2^ = 0.02; Fig. 5). In general, these results show that the assembly and phylogenetic characteristics of widely distributed functional communities were variable in the water and sediment environments of the Zhenhai Bay estuary, and the taxonomic community in the water was most affected by some undetected factors such as hydrology and water environment. Niche breadth was examined to investigate the taxonomic and functional communities that adapted to environmental changes. The niche breadths of the functional communities were significantly (*P* < 0.05) higher than those of the taxonomic communities in the Zhenhai Bay estuary (Fig. S4). These results showed that the functional communities were likely to have higher metabolic flexibility than the taxonomic communities, and the functional community in the water showed higher metabolic flexibility than in the sediment.

### Factors of taxonomic and functional profiles of bacterial communities

To quantify the impact of the terrestrial pollution inputs contributed to the Zhenhai Bay estuary, source-tracking analysis was used to compare the terrestrial pollution inputs contributing to the estuarine bacterial community between the water and sediment. According to the SourceTracker analysis based on microbial taxa, we found that the impact of the terrestrial pollution contributing to the Zhenhai Bay estuarine microbial community showed a significant (*P* < 0.05) difference between water and sediment (Fig. S5). The relative contribution from cropland to water and sediment were 16.56% and 24.23%, respectively, while sewage was estimated to be 13.18% and 1.37%. Particularly, the contribution from unknown sources was estimated to be 49.57% and 74.14% for water and sediment, respectively. This suggests that there are many unknown factors influencing the microbial community in the estuary, which is a highly complex environment.

**Fig 6:**
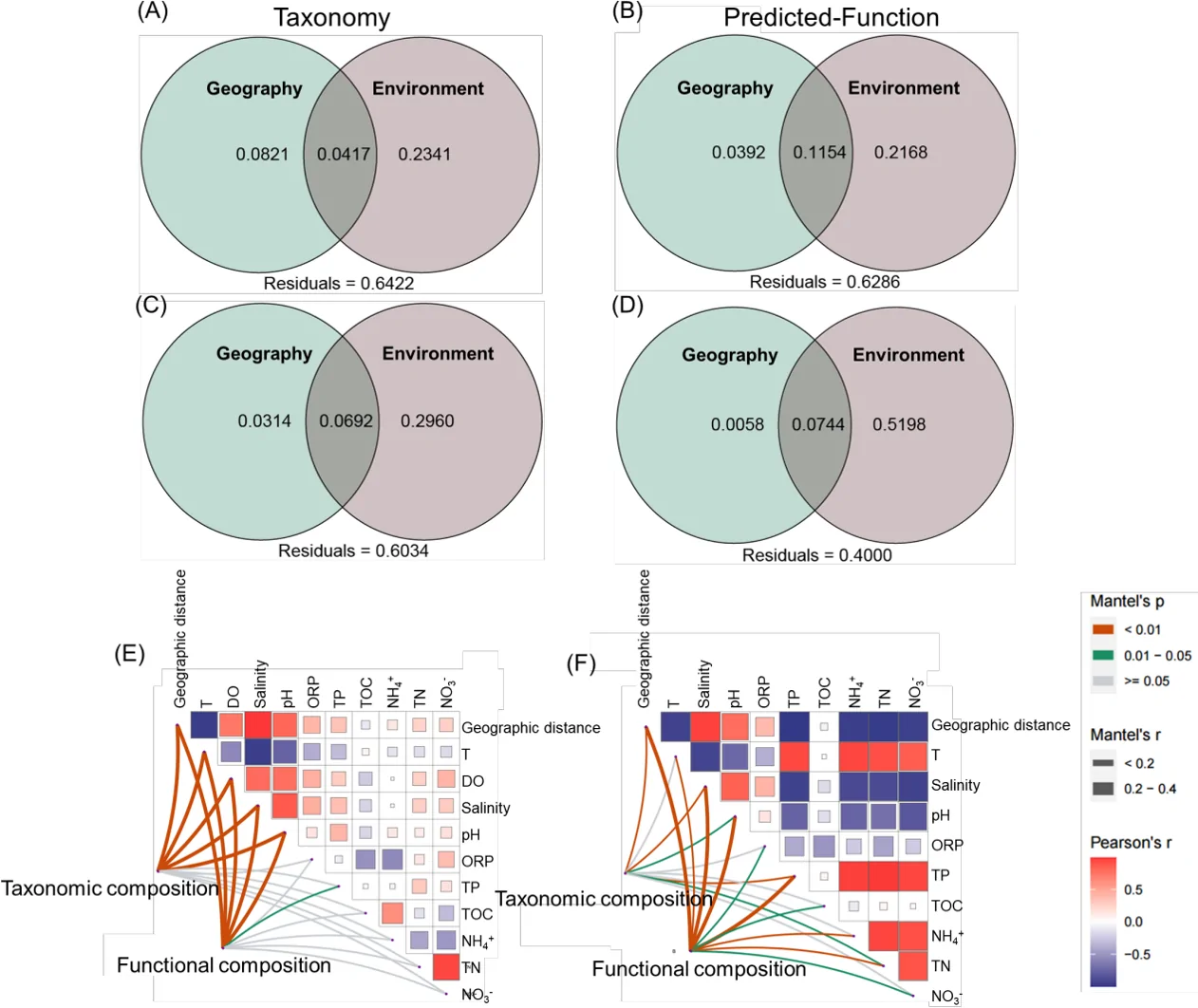
Environmental drivers of bacterial microbial community composition. Contribution of geo-environmental factors to taxonomic and functional compositions as revealed by variation partitioning analysis in water (**A and B**) and sediment (**C and D**), respectively. Environmental drivers of bacterial microbial community composition. Taxa and predicted functions composition were related to each environmental factor by partial Mantel tests in Zhenghai Bay estuary water (**E**) and sediment (**F**). Edge width corresponds to the mantel’s r statistic for the corresponding distance correlations, and edge color denotes the statistical significance based on 9,999 permutations.

In RDA, we found that the measured environmental variables, including temperature (T), salinity, and DO, significantly (*P* < 0.05) impacted variations in the composition of both the taxonomic and predicted functional communities in the water (Fig. S6A and B). The temperature, oxidation-reduction potential, and TP in the sediment were significantly (*P* < 0.05) correlated with taxonomic and predicted functional community compositions (Fig. S6C and D). Moreover, these variables explained 38.23%, 50.19%, 46.61%, and 67.68% of the total variation in the taxonomic and predicted functional community composition of the water and sediment, respectively. This suggested that the mechanisms governing the spatial dynamics of microbial communities in the study area were more complex in the water than in the sediment. Furthermore, the contributions of geographic distance and environmental variables in explaining taxonomic and functional profiles of bacterial communities were quantified by performing variance partitioning (Fig. 6A through D). The total proportions explained by geographic distance and environmental variables were similar for the taxonomic community between water (35.78%), sediment (39.66%), and functional potential in water (37.14%), and the functional potential were the highest in sediment (60%). Moreover, the ratios of geographic distance and environmental variables were dramatically different. The pure explanation ratios of geographic and environmental variables were 8.21% and 23.41% for taxonomic composition in the water, and 3.14% and 29.6% for that in the sediment, respectively, and 3.92% and 21.68% for predicted functional composition in the water, and 0.58% and 51.98% for that in the sediment, respectively. These observations were confirmed by the significant Pearson correlations detected by the Mantel tests (Fig. 6E and F). This indicated that the water microbiome was more often subject to stochastic processes, including dispersal limitations, ecological drift, or due to potential detection limits.

## DISCUSSION

Linking microbial function to taxonomy has been a major challenge in the field of microbial ecology, largely due to the vast diversity and considerable proportion of microbial taxa that remain unknown in natural ecosystems, particularly in estuarine ecosystems. We undertook an analysis of the taxonomic and functional characteristics of bacteria present in estuarine water and sediments using 16S rDNA gene sequencing data. This revealed the unique microbial taxa and their functional attributes. This study yielded pivotal insights into the microbial communities of estuarine habitats, focusing on the distinct microbial profiles of water and sediment, and led to the identification of unique microbial taxa and their associated functional attributes. The use of sequencing data to synthesize broad microbiome patterns provides a valuable insight into the specific functions and survival strategies of microbial taxa that are influenced by the complex estuarine environment. It is imperative to recognize the significance of these changes, particularly in the context of understanding the microbial shifts caused by anthropogenic disturbance.

The composition of the bacterial community depends on the environmental conditions, especially the contrast between water and sediment: aerobic conditions vs anaerobic conditions. *Lactobacillus*, *Romboutsia*, *Turicibacter*, and *Terrisorobacter* were identified as taxonomic indicators of the sediment; except for *Terrisorobacter*, they all belonged to the gut bacteria, indicating that the anthropogenic disturbance played an important role in the Zhenhai Bay sediment. We found that most of the functional indicators of C-, N-, and S-cycling were more enriched in the sediment, suggesting that there may be more active biochemical cycles in sediment than water. It is well known that microbial communities in nature are functionally redundant; many microorganisms have similar functions, and the extinction or dormancy of one species can be compensated for by other functionally similar species (36). The functional redundancy of microbiota has evolved and reflected adaptation to local environmental conditions (37). In the Zhenhai Bay estuary, the water microbiomes exhibited high functional redundancy, which compensated for the disadvantages of low dormancy potential in maintaining ecosystem functions. The type of functional enrichment predicted persistence in stressful environments (54). Additionally, the regression slopes of the water bacterial communities between taxonomic dissimilarity and geographical distance were steeper than those between predicted functional dissimilarity and geographical distance, indicating that the functions of bacterial communities in water were more conservative than their taxonomic compositions. This is consistent with studies reporting that the functional profiles of microbes in natural ecosystems are much more closely related to environmental factors than their taxonomic compositions (37, 48, 55). While microbial communities in the water were likely to be more sensitive to changes in environmental conditions, they were subject to living in a highly dynamic environment (56), which was also supported by the results of the RDA, VPA, and null model. Although the small scale and limited sampling may not be representative of estuarine conditions in such a large, spatially heterogeneous estuary (57–59), this study still provides systematic insights into water and sediment microbial shifts as a result of a minor disturbance. Further sampling, both spatially and temporally across a range of disturbances, would be required to fully understand the microbial dynamics within other large and important estuaries.

Microorganisms are sensitive indicators of estuarine processes because they respond rapidly to dynamic perturbations (3, 11), and their assembly mechanisms are closely linked to changes in community structure, diversity, and the ecological functions they support (25, 60). Although many studies have been carried out to disentangle the mechanisms governing the assembly of complex microbial communities in estuaries (5, 11, 27), there is still no consistent consensus on how the deterministic and stochastic processes shape the microbial community composition. We found that deterministic processes were more important than stochastic processes in the water taxonomic community assembly in the Zhenhai Bay, and stochastic and deterministic processes acted almost in equilibrium in the sediment. Our results were similar to polycyclic aromatic hydrocarbon (PAH) pollution in the Pearl River estuary, which showed that deterministic processes in the highly PAH-polluted water (76%) were much higher than in the low pollution area (7%) and were influenced by deterministic processes (40%) in the sediment (61). Whether it is due to the fact that the Zhenhai Bay estuary was also polluted by PAHs needs to be further studied. However, it is inconsistent with the previous studies that stochastic processes dominated community assembly in estuaries (7, 62). For example, bacterial community assembly in sediments was mainly controlled by deterministic processes, but the dominant assembly processes for water bacteria changed dramatically along the salinity gradient, from homogeneous selection in freshwater to drift in seawater (7). Stochastic processes were also thought to dominate community assembly in sediments along a large river-dominated estuarine shelf in the northern South China Sea (62). The estuarine environment is extremely highly variable and complex, and previous studies have shown that geographical scales, interactions, and environmental gradients significantly influence the balance between deterministic and stochastic processes (11, 17, 61, 63, 64). Notably, most studies rely solely on microbial taxonomic composition to infer the relative importance of stochastic vs deterministic processes (32, 61, 62, 65), ignoring the functional potential carried by microbial taxa. In this study, functional potentials were also used to investigate the relative importance of stochastic vs deterministic processes. However, inconsistent with what has been observed in the global ocean (66), both in water and in sediment, the microbial functional potential was found to be predominantly driven by stochastic factors. Due to functional redundancy in microbial systems, the same functional potential can be carried by many different microbial taxa (37, 67). Only those microbial taxa that perform the required functions and are adapted to the environmental conditions were selected by the ecosystem. Therefore, microbial functional potential is more important than taxa, especially for functionally redundant communities, and the structure of microbial assemblages is constrained to ensure ecosystem multifunctionality and stability. In light of this, we propose that the ecosystem prioritizes the selection of microbial functional potential over taxonomy, with functional redundancy serving as the basis for the stochastic composition of microbial taxa.

Estuaries surrounding highly populated cities are particularly vulnerable to anthropogenic disturbance events (33, 68), which can strongly influence estuarine processes and disrupt the vital ecosystem services that estuaries provide, such as carbon storage, habitat for unique communities, nutrient cycling, and aquaculture (69). Global surveys of microbial diversity have demonstrated that both salinity and environmental type (such as water or sediment) are important factors influencing bacteria (70). In addition to nutrients, river discharge and phytoplankton community composition are also known to drive changes in planktonic estuarine communities (71, 72). However, we found that the percentage of unknown contamination was very high in Zhenhai Bay in both water (49.57%) and sediment (74.14%). Microorganisms have been shown to be sensitive indicators of impact in estuaries due to their rapid growth and response to environmental perturbations such as anthropogenic pollution (73–75). Compared to oceanic plankton assemblages, estuarine plankton can be both more heterogeneous at small spatial scales (76) and experience dynamic temporal changes due to more complex and less predictable hydrographic factors (77). This was also consistent with the null model, which showed that the taxonomic community in the water of Zhenhai Bay, which is a small-scale estuary. Additionally, whether it is the whole community or the indicators, it is the functional potential that is more relevant to the environment, especially for microbiomes in sediment than in water (Fig. 2C and D; Fig. S5). One interpretation is that the assembly of bacterial communities is based on functional genes rather than species (36). Moreover, the functional potential of microbiota has evolved and reflected adaptation to local environmental conditions (37). This is consistent with studies reporting that microbial functional profiles in natural ecosystems (e.g., oceans and soils) are much closer to environmental factors than to taxonomic composition (48, 66). Additionally, evidence has shown that the identity of the taxa encoding each function can vary substantially across space or time with little effect on the function, and this taxonomic variability is often attributed to result from ecological drift between equivalent organisms (37, 55). Further sampling, both spatially and temporally across a range of disturbances, would be necessary to fully understand the microbial dynamics within the complex estuarine ecosystems.

### Conclusion

Understanding the processes, mechanisms, and factors underlying biodiversity, loss of biodiversity, and associations with ecosystem functions is crucially needed and relevant for all ecosystems worldwide. In summary, the integrated analysis of geochemical parameters, terrestrial pollution, and bacterial communities revealed relationships between bacterial communities and the environment in water and sediments. This was a detailed study that considered the effects of terrestrial pollution on the microbiology of estuarine water and sediment, and the results showed that the dynamics of estuarine microbial communities are much more complex than expected. This subject remains largely unexplored, requiring the use of a range of technological tools and other estuarine systems to facilitate a more comprehensive understanding.

## SUPPLEMENTAL MATERIAL

Supplemental material spectrum.02249-25-s0001.docx Fig. S1–S6; Tables S1–S3.

ASM does not own the copyrights to Supplemental Material that may be linked to, or accessed through, an article. The authors have granted ASM a non-exclusive, world-wide license to publish the Supplemental Material files. Please contact the corresponding author directly for reuse.
