# Supplementary material for: Insights into the assembly mechanisms, co-occurrence patterns, and functional roles of bacterial community in an estuary system
Source: Microbiol Spectr. 2026 Aug 17;14(10):e02249-25. doi: 10.1128/spectrum.02249-25 (PMC13688845; doi:10.1128/spectrum.02249-25)
Supplement: Supplementary file 1 [file spectrum.02249-25-s0001.docx]

**Supplementary Figures and Tables**

**Supplementary Figure 1** Differences in the Shannon diversity (A), Richness (B), Simpson diversity (C) and Chao1 diversity (D) of bacterial communities between the water and sediment in Zhenghai Bay estuary.

**Supplementary Figure 2** The microbiome of various between water and sediment in Zhenghai Bay estuary at the phylum level (A) and genus level (B), respectively.

**Supplementary Figure 3** Bar charts showing functional distributions of dominant bacteria (abundance top 15) for the Zhenghai Bay estuary in water and sediment (A) samples based on sequence data, and the relative abundances of C-, N- and S-cycling function between water and sediment (B).

**Supplementary Figure 4** The niche width (log10 transformed) of bacterial taxonomy and predicted functions composition between water and sediment in Zhenghai Bay estuary.

**Supplementary Figure 5** Source–tracking analysis showing the relative contributions of terrestrial pollutions to the water and sediment in Zhenghai bay estuary (A), and the variation of cropland (B), sewage (C), and unknown (D) sources along the sampling sites. “Unknown” includes the percentage of contributions not able to be attributed to a specific source. Different capital letters mean a statistical significance (p < 0.05) between water and sediment within the same terrestrial pollution, and different small letters mean a statistical significance (p < 0.05) among different terrestrial pollutions within the same habitat.

**Supplementary Figure 6** Redundancy analysis based on the water taxonomy (A), water predicted-function (B), sediment taxonomy (C) and sediment predicted-function (D) community structure and physicochemical variables. The dashed arrows indicate that environmental factors did not significantly affect the dissimilarity of taxonomy and predicted-functional communities at the OTU level. NO_3_^−^, NH_4_^+^, TN, TP, DO, T and ORP represent NO3−-N, NH4+-N, total nitrogen, total phosphorus, dissolved oxygen, temperature and oxidation-reduction potential, respectively.

.**Supplement Table1** Physiochemical properties of water and sediment in Zhenghai Bay estuary

| Sampling sites | | T | DO | Salinity | pH | ORP | TP | TOC | NH_4_^-^ | NO_3_^-^ | TN |
| --- | --- | --- | --- | --- | --- | --- | --- | --- | --- | --- | --- |
| Water | S1 | 31.0±0.1 | 4.28±0.08 | 11.67±0.04 | 7.29±0.02 | 116.17±8.33 | 2.1±0.05 | 4.87±1.23 | 5.43±1.46 | 1.02±0.21 | 6.91±0.09 |
|  | S2 | 31.6±0.1 | 4.83±0.06 | 11.48±0.55 | 7.39±0.01 | 94.33±1.63 | 3.96±0.14 | 6.23±0.62 | 19.75±5.34 | 1.17±0.05 | 7.20 ±0.56 |
|  | S3 | 30.4±0.2 | 4.21±0.02 | 18.24±0.08 | 7.45±0.01 | 93.43±2.97 | 5.45±0.48 | 6.45±0.41 | 15.02±0.61 | 0.353±0.33 | 5.97±0.77 |
|  | S4 | 30.4±0.1 | 5.2±0.09 | 23.85±0.17 | 7.64±0.01 | 105.77±2.83 | 3.8±0.08 | 5.39±0.62 | 20.84±3.8 | 1.23±0.28 | 4.67±0.52 |
|  | S5 | 30.2±0.2 | 5.31±0.11 | 25.4±0.06 | 7.67±0.01 | 113.1±5.62 | 3.52±0.14 | 4.63±1.22 | 8.49±0.14 | 1.38±0.59 | 6.57±0.41 |
|  | S6 | 29.8±0.1 | 5.36±0.07 | 28.13±0.04 | 7.72±0.01 | 106.17±7.46 | 5.2±0.14 | 5.39±0.1 | 7.46±0.32 | 1.94±0.11 | 9.45±0.91 |
|  | S7 | 30.1±0.1 | 5.36±0.1 | 29.4±0.13 | 7.74±0.01 | 112.7±6.89 | 4.12±0.28 | 4.6±0.5 | 6.71±0.34 | 0.567±0.45 | 5.60±0.31 |
|  | S8 | 29.5±0.1 | 5.41±0.16 | 30.33±0.03 | 7.75±0.01 | 123.87±7.58 | 6.33±0.32 | 4.28±1.06 | 6.47±0.58 | 1.27±0.29 | 6.43±0.12 |
|  | S9 | 29.2±0.1 | 5.12±0.14 | 32.74±0.01 | 7.63±0.3 | 121.2±9.5 | 3.62±0.03 | 4.89±0.77 | 7.03±0.26 | 1.81±0.67 | 9.63±0.22 |
|  | S10 | 28.7±0.1 | 5.31±0.05 | 32.96±0.02 | 7.81±0.01 | 103.77±2.48 | 4.01±0.17 | 6.91±0.53 | 31.27±7.84 | 1.59±0.62 | 4.47±0.33 |
| Sediment | S1 | 30.0±0.1 | NA | 11.67±0.04 | 7.11±0.02 | 116.17±8.33 | 708.5±3.67 | 48.7±1.48 | 56.8±2.89 | 12.4±0.23 | 986.5±5.73 |
|  | S2 | 30.6±0.1 | NA | 11.48±0.55 | 7.34±0.01 | 94.33±1.63 | 682.4±2.45 | 62.28±2.67 | 54.7±0.89 | 9.8±0.11 | 973.3±3.58 |
|  | S3 | 30.4±0.1 | NA | 18.24±0.08 | 7.46±0.01 | 93.43±2.97 | 654.3±3.89 | 64.51±3.56 | 49.3±1.22 | 8.9±0.24 | 943.3±4.37 |
|  | S4 | 29.4±0.1 | NA | 23.85±0.17 | 7.62±0.01 | 105.77±2.83 | 627.4±4.56 | 53.9±1.08 | 47.2±1.45 | 8.7±0.56 | 918.4±2.76 |
|  | S5 | 29.2±0.2 | NA | 25.4±0.06 | 7.65±0.01 | 113.1±5.62 | 598.6±2.38 | 46.29±2.77 | 45.1±0.77 | 7.87±0.52 | 888.9±7.21 |
|  | S6 | 29.8±0.1 | NA | 28.13±0.04 | 7.02±0.01 | 106.17±7.46 | 579.7±4.78 | 53.87±1.09 | 42.5±2.90 | 6.95±0.89 | 838.3±5.93 |
|  | S7 | 29.1±0.1 | NA | 29.4±0.13 | 7.04±0.01 | 112.7±6.89 | 558.8±1.04 | 46.01±3.67 | 39.6±2.19 | 6.04±0.66 | 766.5±3.54 |
|  | S8 | 29.5±0.1 | NA | 30.33±0.03 | 7.15±0.01 | 123.87±7.58 | 508.6±6.66 | 42.78±2.88 | 37.4±0.99 | 5.92±0.76 | 695.2±3.87 |
|  | S9 | 28.2±0.1 | NA | 32.74±0.01 | 7.33±0.3 | 121.2±9.5 | 482.1±3.67 | 48.87±3.97 | 34.3±0.42 | 5.87±0.44 | 643.4±2.54 |
|  | S10 | 28.7±0.1 | NA | 32.96±0.02 | 7.41±0.01 | 103.77±2.48 | 461.4±4.08 | 69.08±3.88 | 22.1±0.66 | 5.1±0.73 | 620.9±2.31 |

Note: Data are presented as mean ± standard error (n=3).

**Supplement Table2** Topological properties of the empirical molecular ecological networks in comparison to the random networks.

| Network Indexes | **Empirical networks** | |  | **Random networks^a^** | |
| --- | --- | --- | --- | --- | --- |
|  | Water | Sediment |  | Water | Sediment |
| Similarity threshold | 0.89 | 0.93 |  | / | / |
| Total nodes | 239 | 410 |  | / | / |
| Total edges | 976 | 5157 |  | / | / |
| R^2^ of power-law | 0.843 | 0.806 |  | / | / |
| Average degree (avgK) | 5.191 | 25.041 |  | / | / |
| Average clustering coefficient (avgCC) | 0.265 | 0.413 |  | 0.052 ±0.007 | 0.208 ±0.006 |
| Harmonic geodesic distance (HD) | 4.011 | 3.074 |  | 3.151±0.026 | 2.334±0.008 |
| Modularity | 0.589 | 0.403 |  | 0.399±0.005 | 0.130±0.003 |
| Module number | 29 | 17 |  | / | / |
| Negative edge number | 33 | 454 |  | / | / |
| Positive edge number | 943 | 4703 |  | / | / |

a Given are the mean ± standard derivations that generated from 100 times of randomly rewired networks.

**Supplement Table3** Scientific classification and network topological properties of hub nodes in networks

| **Habitats** | **OTU classification** | **Zi** | **Pi** | **Degree** | **Clustering Coefficient** |
| --- | --- | --- | --- | --- | --- |
| Water | OTU_3653(p_Planctomycetes; c_Planctomycetacia; o_Isosphaerales; f_Isosphaeraceae; g_Aquisphaera) | -0.41 | 0.64 | 5 | 0.1 |
|  | OTU_520(p_Chloroflexi; c_Ktedonobacteria; o_Ktedonobacterales; f_Ktedonobacteraceae; g_HSB_OF53-F07) | 3.92 | 0.48 | 15 | 0.14 |
|  | OTU_878(p_Proteobacteria; c_Alphaproteobacteria; o_Rhizobiales; f_Xanthobacteraceae; g_Pseudolabrys) | 3.88 | 0.21 | 17 | 0.16 |
|  | OTU_173(p_Chloroflexi; c_KD4-96; o_Unclassified; f_ Unclassified; g_ Unclassified) | 3.23 | 0.27 | 47 | 0.24 |
|  | OTU_44(p_Proteobacteria; c_Deltaproteobacteria; o_Myxococcales; f_Sandaracinaceae; g_Unclassified) | 3.21 | 0 | 15 | 0.22 |
|  | OTU_195(p_Proteobacteria; c_Deltaproteobacteria; o_Myxococcales; f_Archangiaceae; g_Anaeromyxobacter) | 3.07 | 0 | 6 | 0.07 |
|  | OTU_363(p_Actinobacteria; c_Actinobacteria; o_Frankiales; f_Acidothermaceae; g_Acidothermus) | 3.01 | 0.14 | 41 | 0.29 |
|  | OTU_84(p_Proteobacteria; c_Gammaproteobacteria; o_Unclassified; f_ Unclassified; g_ Unclassified) | 2.92 | 0.12 | 15 | 0.16 |
|  | OTU_310(p_Nitrospirae; c_Thermodesulfovibrionia; o_Unclassified; f_ Unclassified; g_ Unclassified) | 2.57 | 0.1064 | 36 | 0.38 |
|  | OTU_1428(p_Chloroflexi; c_KD4-96; o_Unclassified; f_ Unclassified; g_ Unclassified) | 2.55 | 0 | 11 | 0.07 |
|  | OTU_2532(p_Acidobacteria; c_Subgroup_6; o_Unclassified; f_ Unclassified; g_ Unclassified) | 2.55 | 0 | 11 | 0.13 |
| Sediment | OTU_913(p_Proteobacteria; c_Deltaproteobacteria; o_Desulfarculales; f_Desulfarculaceae; g_Desulfatiglans) | 4.72 | 0.25 | 14 | 0.09 |
|  | OTU_132(p_Bacteroidetes; c_Bacteroidia; o_Bacteroidales; f_Prevotellaceae; g_Prevotella_9) | 4.34 | 0.54 | 145 | 0.22 |
|  | OTU_506(p_Acidobacteria; c_Subgroup_17; o_Acidobacteria_bacterium_RBG_16_70_10; f_ Unclassified; g_ Unclassified) | 3.98 | 0 | 15 | 0.11 |
|  | OTU_218(p_Chloroflexi; c_Ktedonobacteria; o_Ktedonobacterales; f_JG30-KF-AS9; g_Unclassified) | 3.63 | 0 | 14 | 0.16 |
|  | OTU_44(p_Proteobacteria; c_Deltaproteobacteria; o_Myxococcales; f_Sandaracinaceae; g_ Unclassified) | 3.46 | 0.33 | 196 | 0.21 |
|  | OTU_371(p_Proteobacteria; c_Gammaproteobacteria; o_Steroidobacterales; f_Woeseiaceae; g_Woeseia) | 3.22 | 0.37 | 193 | 0.21 |
|  | OTU_54(p_Proteobacteria; c_Gammaproteobacteria; o_Steroidobacterales; f_Woeseiaceae; g_Woeseia) | 3.22 | 0.35 | 190 | 0.23 |
|  | OTU_181(p_Proteobacteria; c_Gammaproteobacteria; o_Ectothiorhodospirales; f_Thioalkalispiraceae; g_SS1-B-09-64) | 3.03 | 0 | 78 | 0.36 |
|  | OTU_457(p_Proteobacteria; c_Deltaproteobacteria; o_Desulfuromonadales; f_Sva1033; g_ Unclassified) | 3.03 | 0 | 78 | 0.33 |
|  | OTU_307(p_Proteobacteria;c_Gammaproteobacteria;o_Gammaproteobacteria_Incertae_Sedis;f_Unclassified;g_Unclassified) | 2.95 | 0.28 | 167 | 0.27 |
|  | OTU_84(p_Proteobacteria; c_Gammaproteobacteria; o_Unclassified; f_ Unclassified; g_ Unclassified) | 2.95 | 0.33 | 176 | 0.22 |
|  | OTU_173(p_Chloroflexi; c_KD4-96; o_Unclassified; f_ Unclassified; g_ Unclassified) | 2.93 | 0 | 12 | 0.28 |
|  | OTU_561(p_Acidobacteria; c_Subgroup_17; o_Unclassified; f_ Unclassified; g_ Unclassified) | 2.92 | 0.26 | 164 | 0.26 |
|  | OTU_3724(p_Proteobacteria; c_Deltaproteobacteria; o_Desulfobacterales; f_Desulfobulbaceae; g_ Unclassified) | 2.77 | 0.02 | 74 | 0.37 |
|  | OTU_406(p_Chloroflexi; c_Anaerolineae; o_Ardenticatenales; f_ Unclassified; g_ Unclassified) | 2.77 | 0.12 | 78 | 0.27 |
|  | OTU_998(p_Acidobacteria; c_Subgroup_9; o_Unclassified; f_ Unclassified; g_ Unclassified) | 2.72 | 0.19 | 9 | 0.27 |
|  | OTU_213(p_Chloroflexi; c_Anaerolineae; o_Anaerolineales; f_Anaerolineaceae; o_Unclassified) | 2.61 | 0.10 | 74 | 0.34 |
|  | OTU_1474(p_Chloroflexi; c_Anaerolineae; o_Unclassified; f_ Unclassified; g_ Unclassified) | 1.23 | 0.66 | 15 | 0.37 |
|  | OTU_1946(p_Firmicutes; c_Bacilli; o_Lactobacillales; f_Enterococcaceae; g_Enterococcus) | -0.64 | 0.66 | 3 | 0.33 |
|  | OTU_9424(p_Proteobacteria; c_Deltaproteobacteria; o_Desulfobacterales; f_Desulfobulbaceae; g_Desulfobulbus) | 0.59 | 0.66 | 10 | 0.57 |
|  | OTU_25573(p_Firmicutes; c_Clostridia; o_Clostridiales; f_Ruminococcaceae; g_Ruminococcaceae_UCG-005) | 0.18 | 0.65 | 12 | 0.12 |
|  | OTU_3158(p_Firmicutes; c_Clostridia; o_Clostridiales; f_Lachnospiraceae; g_[Eubacterium]_xylanophilum_group) | -0.64 | 0.64 | 5 | 0 |
|  | OTU_519(p_Proteobacteria; c_Deltaproteobacteria; o_Syntrophobacterales; f_Syntrophobacteraceae; g_ Unclassified) | 0.23 | 0.62 | 12 | 0.48 |
